# Antiphospholipid Antibodies: From General Concepts to Its Relation with Malignancies

**DOI:** 10.3390/antib5030018

**Published:** 2016-08-02

**Authors:** José A. Gómez-Puerta, Gerard Espinosa, Ricard Cervera

**Affiliations:** 1 Grupo de Inmunología Celular e Inmunogenética y Grupo de Reumatología, Universidad de Antioquia, Medellín 05004, Antioquia, Colombia; 2 Consultor de Reumatología, Dinámica IPS, Medellín 050015, Antioquia, Colombia; 3 Department of Autoimmune Diseases, Hospital Clínic, Villarroel, 170, Barcelona 08036, Catalonia, Spain

**Keywords:** antiphospholipid antibodies, lupus anticoagulant, anticardiolipin antibodies, cancer, malignancies, catastrophic antiphospholipid syndrome

## Abstract

Antiphospholipid syndrome (APS) is an adquired autoimmune pro-thrombotic disease characterized by arterial and/or venous thrombosis and/or fetal losses associated with the persistent presence of antiphospholipid antibodies (aPL) detectable by solid phase assays (anticardiolipin (aCL) and anti-β2 glycoprotein I, β2GPI) and/or functional coagulation test (lupus anticoagulant (LA)). Most patients with typical APS manifestations have the presence of one or more of conventional aPL, but, some patients might exhibit clinical features related with APS but with persistent negative determinations of “classic” aPL (seronegative APS). Expanding the network of autoantibodies in patients highly suspected of having APS but who have normal results from a conventional test using new antibodies (i.e., phosphatidylserine/prothrombin and β2GPI domain 1) would increase the diagnosis. Thrombosis is one of the leading causes of death among patients with cancer, representing up to 15% of all deaths. Cancer increases the risk of thrombosis and chemotherapy is further associated with a higher risk of thrombosis. In addition, aPL may contribute to an increased risk of thrombosis in patients with malignancies, although the levels do not seem to reflect their pathogenicity. Several malignancies, particularly hematological and lymphoproliferative malignancies, may indeed be associated with the generation of aPL but do not necessarily enhance the thrombophilic risk in these patients.

## 1. Introduction

Antiphospholipid syndrome (APS) is defined by the presence of arterial and venous thromboses and pregnancy morbidity (miscarriages, fetal deaths, premature births), in the company of antiphospholipid antibodies (aPL); namely, lupus anticoagulant (LA), anticardiolipin antibodies (aCL), or anti-β2 glycoprotein-I (anti-β2GPI) antibodies. APS can occur in patients having neither clinical nor laboratory evidence of another definable condition (primary APS), or it may be associated with other diseases, mainly systemic lupus erythematosus (SLE), and occasionally with other autoimmune conditions (Sjögren syndrome, systemic vasculitis, rheumatoid arthritis, among others), infections, drugs, and malignancies [1].

Despite the prevalence of aPL in general population is up to 5%, only a small proportion of patients develop APS. Some epidemiological studies estimates that the incidence of APS is around 5 new cases per 100,000 persons per year and the prevalence around 40–50 cases per 100,000 persons [2]. The prevalence increases in the elderly and in those with chronic disease. Very recently, a population-based cohort was conducted in Germany (Gutenberg Health Study) including 5000 subjects (2540 men, 2460 women) from April 2007 to October 2008 [3]. aCL, anti-β2GPI and anti- β2GPI domain 1 were measured in 4977 subjects. The authors found a strong age-dependent increase of both aCL and anti-β2GPI IgM, while aPL IgG titers were stable or tended to decrease with age [3].

In 2011, an international group, APS ACTION (AntiPhospholipid Syndrome Alliance For Clinical Trials and InternatiOnal Networking), gathered with the aim of planning several studies on aPL-related syndromes. Its primary mission involves the prevention, treatment, and cure of aPL-associated clinical manifestations through high-quality, multicentre, and multidisciplinary clinical research. Recently, the APS ACTION group published a literature review focused on the prevalence of aPL in the general population for 4 different outcomes of APS: stroke, myocardial infarction (MI), deep vein thrombosis (DVT) and pregnancy morbidity. APS action group estimated that aPL are positive in approximately 13% of patients with stroke, 11% with MI, 9.5% of patients with DVT and 6% of patients with pregnancy morbidity [4].

One of the pivotal studies on APS was conducted by The Euro-Phospholipid Group. This International group of experts from 13 European countries analyzed the prevalence of the most relevant clinical and immunological features in a cohort of 1,000 APS patients. Stroke and transient ischemic attacks were the most common arterial manifestations (19.8%, 11.1% respectively), followed by leg ulcers (5.5%), MI (5.5%), and amaurosis fugax (5.4%). Regarding venous events, the most frequent features were: DVT (38.9%), pulmonary embolism (14.1%), and superficial thrombophlebitis (11.7%). Other clinical manifestations included thrombocytopenia (29.6%), *livedo reticularis* (24.1%), heart valve lesions (14.3%), hemolytic anemia (9.7%) and epilepsy (7%) among others [5].

One uncommon, but often lethal variant of APS, characterized by a rapid and progressive thrombosis (mainly small vessel trombosis) is known as catastrophic APS (CAPS) [6]. Fortunately the prevalence of the catastrophic APS is rare (<1% of all cases of APS) but its potentially fatal outcome emphasizes its significance in clinical practice. In order to summarize all the published case reports as well as the new diagnosed cases from all over the world, an international registry of patients with catastrophic APS (“CAPS Registry#x201D;) was created in 2000 by the European Forum on Antiphospholipid Antibodies. Currently, CAPS registry includes clinical, laboratory and therapeutic data of around 500 cases. This registry can be freely checked on the Internet https://ontocrf.grupocostaisa.com/es/web/caps/home.

## 2. aPL Antibodies

aPL antibodies are a heterogenous group of autoantibodies directed against anionic phospholipids or protein-phospholipid complexes, measured in solid phase immunoassays such as aCL or as an activity (functional assays) which prolongs phospholipid-dependent coagulation assays, the so-called LA. There are three well described and validated aPL antibodies included in the current revised classification criteria (“Sydney criteria”) [7], including aCL (IgG and IgM), LA and β2GPI (IgG and IgM). Accoding to revised classification criteria, aCL antibodies are considered positive when they are present in serum or plasma at medium or high titers on two or more occasions, at least 12 weeks apart, measured by a standardized ELISA technique. LA is positive when it is present in plasma, on two or more occasions at least 12 weeks apart, detected according to the guidelines of the International Society on Thrombosis and Hemostasis. Finally, Anti-β2GP1 antibodies are considered positive in serum or plasma, on two or more occasions, at least 12 weeks apart, measured by a standardized ELISA technique. Some other autoantibodies directed against anionic phospholipids or serological assays that detect antibodies to coagulation proteins have been reported during the last years [8]. However, not all of them have been replicated in other groups or have been standardized using conventional techniques (Table 1).

APS patients nor only have the presence of aPL antibodies, but also a wide variety of autoantibodies in secondary APS patients, including antinuclear antibodies, anti-dsDNA antibodies and extractable nuclear antigen antibodies, among others. The most common immunological features in APS patients are collected in Table 2.

## 3. Seronegative APS

Seronegative APS is defined as patients with typical manifestations suggestive of APS (i.e., *livedo reticularis*, recurrent pregnancy losses, DVT or thrombocytopenia) but who have tested persistently negative for conventional aPL on several occasions. The term seronegative APS was quoted for the first time by Hughes and Khamashta [9]. Some potential explanations for seronegative APS include (1) antibody consumption during an acute thrombotic episode; (2) transient negativity of previously positive aPL patients (unlikely); and (3) a more realistic one: antibodies to the heterogeneous aPL family against protein and protein-bound phospholipids which have not been identified to date. The most promising of “non-classic” aPL are antibodies to phospholipid-protein complexes (vimentin/cardiolipin complex), antibodies against phospholipid-binding plasma proteins (prothrombin (PT), protein C, protein S, annexin V, and domains of β2GPI) [10]; phospholipid-protein complexes (vimentin/cardiolipin complex); and anionic phospholipids other than cardiolipin (phosphatidylserine (PS), phosphatidylinositol) [11] and antibodies to the complex PS/PT [12].

Expanding the network of autoantibodies in patients with normal results from a classic test (aCL, LA and/or β2GPI) using new antibodies (i.e., PT/PS and β2GPI domain 1) in patients with suspected APS would increase the diagnostic capability of detection of new cases of APS formerly labeled as “seronegative” cases [13]. Recent reports have shown that anti β2GPI domain 1 antibodies might achieve a specificity as high as 99.5% for patients with APS and thrombosis events [14].

## 4. aPL and Malignancies

It is known as Trosseau’s syndrome the association between neoplastic disease and a thromboembolic disorder made by Armand Trousseau in 1865 [15]. During decades, the relationship between thrombosis and cancer was well documented. During the last 40 years, several case reports of aCL in patients with thrombotic events and malignant conditions, including hematological, lymphoproliferative disorders and solid tumors have been published. The increasing knowledge of aPL in the pathogenesis of vascular occlusions established a close link between aPL and malignancies.

There has been experimental work demonstrating tumor growth with agents activating blood coagulation and regression with coagulation inhibitors. Fibrin generation has also been associated with accelerated tumor growth and tumor cells themselves may be responsible for the production of compounds resulting in this mechanism of thrombosis [16].

Tumoral cells activate coagulation system through different pathways, interacting with clot cells, platelets and fibrinolytic systems to generate thrombin. In addition, some other endothelial factors, such as fibrin and tissue factor might play a role in the clotting formation mediated via fibrin deposition and platelet activation [17].

Several mechanisms have been suggested to explain the association between aPL and cancer including the following: (1) production of autoantibodies as a response to tumor antigens; (2) secretion of aCL from tumor cells; and (3) production of monoclonal immunoglobulins with LA and aCL activities [18].

In addition, some other clinical factors contribute to the risk of thrombosis including immobilization or intravenous catheters. Furthermore, the risk of thrombosis appears to be highest during the initial hospitalization and onset of chemotherapy, as well as at the time of disease progression [19].

Information about the association between aPL and malignancies (solid tumors and hematological malignancies) are heterogeneous and some, but not all, included information regarding clinical features. Different series of patients with aPL and malignancies are summarized on Table 3.

One of the main studies in the field, was conducted in early 90’s in Montepellier, France [20]. The study included 1014 patients who were tested at entry for aCL, been carcinoma was the most frequently associated disease. Only 7.1% of subjects were positive for aPL. Among them, 14 had a history of carcinoma, 9 had active malignant and 5 were in remission. The main related malignancies found were prostatic adenocarcinoma, breast carcinoma, ovarian carcinoma, and colon adenocarcinoma.

Another study evaluated the prevalence of aPL in 216 consecutive patients admitted with a biopsy/cytology proven neoplastic disease [21]. Additionally, the study included as a control group 88 age-matched healthy subjects. aPL were more prevalent among cancer patients (22%) in comparison with control group (3%). Among cancer patients, thrombotic rates were higher in aCL-positive patients (13 out of 47, 28%) than in aCL-negative patients (24/169, 14%).

A German group, retrospectively studied the presence of aPL and thrombotic manifestations in a cohort of 58 patients with previous history of neoplasia (39 solid tumours and 19 hematologic/lymphoproliferative disease) [22]. LA was positive in 46% of patients, IgG aCL in 41%, IgM aCL in 64%, and 55% of patients had elevated levels of both. Of the patients with solid tumors, 18/39 (46%) patients had thromboembolic features of APS. Of the patients with hematologic and lymphoproliferative malignancies, only 6/19 (32%) suffered from thromboembolic events.

Some years ago, we performed a literature review of cases with aPL related with solid tumors, lymphoproliferative and hematological malignancies [18]. Given the heterogeneity of information, a wide list of neoplastic disorders were identified. B-cell lymphoma, splenic lymphoma, chronic myeloid leukemia, and non-Hodgkin’s lymphoma were the most common hematologic disorders. Regarding solid tumors, renal cell carcinoma, primary tumor with unknown origin, lung adenocarcinoma and breast carcinoma were the main solid tumors related with aPL. In that series, 29 out of 120 cases of malignancy were diagnosed after the thrombotic manifestation of APS and in 41 cases, the diagnosis of both conditions (APS and cancer) was made at the same time.

A big cohort of cytology/histologically-confirmed solid tumor patients with an active disease with a new diagnosis of venous thromboembolism (VTE) was recently published [23]. In addition, two age-sex matched groups were included (one group of outpatients with diagnosis of solid tumors and no history of VTE and one group of healthy individuals without previous thrombotic events, history of abortions or autoimmune disease). Finally, the study included 258 patients with cancer and VTE, 142 patients with cancer without VTE and 258 healthy controls. aPL antibodies (aCL, LA and B2GPI) were measured in the first 72 hours after VTE and at least 12 weeks after the first in aPL positive patients and healthy controls. aPL were more prevalent in patients with cancer and VTE (21 out of 258; 8.1%) compared to cancer patients VTE negative (2 out of 142; 1.4%) and healthy subjects (2 out of 258; 0.8%). LA and aCL IgG were the most-frequent aPL, followed by β2GPI IgG antibodies. The authors concluded that in comparison with cancer patients without VTE and healthy individuals, cancer patients with VTE had an elevated prevalence of aPL. In addition, it was suggested that the presence of aPL may identify a subset of cancer patients who are at high risk of developing thrombotic complications. For instance, those patients with persistent aPL positivity, especially those with triple positivity (aCL, LA and β2GPI) need a closer monitoring due to a higher risk of new episodes of thrombosis, even in patients under anticoagulation treatment.

Since the former cited studies place a focus on “classic” antibodies included on Sydney criteria, it is possible that some other cases of malignancy-associated thrombosis might be attributed to “novel” aPL. Whether these subgroups of patients represent a different clinical outcome or a worse prognosis requires further analysis.

## 5. Catastrophic APS

As we cited previously, Catastrophic APS is an uncommon form of presentation of APS, in the majority of case characterized by severe thrombotic complications predominantly affecting small vessels of organs, however, some patients can developed large vessel involvement as occurs in classic forms of APS [7]. Of the 488 cases included so far in the CAPS registry (https://ontocrf.grupocostaisa.com/es/web/caps/home) 50 (10.2%) patients had malignancies; 28 (56%) were female and 22 (44%) were male. The mean age was 46.9 years (SD 22 years). Of the patients, 3 (6%) had SLE, and 44 (88%) had a primary APS. LA were detected in 40 patients (80%), IgG aCL in 35 patients (70%), and IgM aCL in 23 patients (46%). Almost half of patients had thrombocytopenia (42% of cases).

Combined therapy with anticoagulation, steroids, plasma exchange and/or intravenous immunoglobulins is the standard treatment of patients with CAPS. Despite that treatment, survival rate of patients with CAPS still poor. The outcome of patients with CAPS is worse in the presence of an additional malignancy than when no malignancy is present. Only 38% of CAPS patients with malignancies recovered in comparison with 64% of patients without malignancies (*p* < 0.05). Treatment modalities, however, did not differ significantly between these patients. Only around 40% of CAPS patients with malignancies improved. This may be due to the additional presence of the malignancy and to the older age of the patients. Other potential confounding factors such as concomitant chemotherapy treatment, cancer stage, disease duration, comorbidities, among others were not assessed.

## 6. Conclusions

aPL are a heterogenous group of autoantibodies directed at phospholipid binding proteins. The “classic” aPL include LA, aCL and anti-β2GP1 antibodies; however, some autoantibodies directed against anionic phospholipids or coagulation proteins have been reported during the last years. The presence of aPL may contribute to an increased risk of thrombosis in patients with malignancies, although the levels do not seem to reflect their pathogenicity. Probably, the persistence of aPL over time, and the combination of two or more aPL, has more specific weight on the risk of thrombosis during follow-up.

According to previous data, it is desirable to perform a complete search of aPL in those patients with cancer and thrombosis. Conversely, it also important, rule out a neoplastic disorder in those patients with APS (either classic forms or CAPS) with a new episode of thrombosis despite an adequate range of anticoagulation.

In the future, it will be desirable to identify different profiles of patients with APS, according their thrombotic and non-thrombotic features and according their aPL panel. Distinguishing the different profiles of patients will, no doubt, present an opportunity to treat in a better way patients suffering from APS, including those patients with malignancies related with aPL.

## Figures and Tables

**Table 1:** Criteria and non-criteria antiphospholipid antibodies (aPL) antibodies.

**Criteria aPL**
aCL IgG and IgM
Anti-β2GPI IgG and IgM
LA
**Other non-criteria aPL**
aCL IgA
Anti-β2GPI IgA
Anti-annexin A2
Anti vimentin/cardiolipin complex
Anti-annexin A5
Antiphosphatidylethanolamine
Antiphosphatidylinositol
Anti PT/PS *
Anti-β2GPI Domain I *

* New promising criteria aPL.

**Table 2:** Most common autoantibodies in the antiphospholipid syndrome (APS), according to the “Euro-Phospholipid Project” (including patients with Primary APS and associated APS, mainly systemic lupus erythematosus (SLE)) [5].

Autoantibody	%
aCL	87.9
IgG and IgM aCL	32.1
IgG aCL alone	43.6
IgM aCL alone	12.2
LA	53.6
LA alone	12.1
LA and aCL	41.5
ANA	59.7
Anti-dsDNA	29.2
Anti-Ro/SS-A	14
Anti-La/SS-B	5.7
Anti-RNP	5.9
Anti-Sm	5.5
Rheumatoid factor	7.8

No information about β2GPI antibodies was available.

**Table 3:** Series of patients with Malignancies and aPL.

Author (year) REF	No. Patients	Mean Age (years)	Female Gender (%)	Solid Tumors	Haematological Neoplasms	aPL	APS	Thrombotic Manifestations
Gómez-Puerta et al. (2006) [18]	120	56	48	Renal cell carcinoma 6% Primary unknown origin 5% Lung adenocarcinoma 5% Breast cancer 5%	B Cell lymphoma 8% Spleen lymphoma 7% Chronic myeloid leukemia 5%	LA 67% aCL (67%) (54 aCL IgG and 20 aCL IgM, B2GP1 6%)	Primary APS in 22 (18%) patients *	Thrombotic manifestations 71% APS Sapporo criteria in 21%
Zuckerman et al. (1995) [21]	216	67	45	Colerectal cancer 17% Lung carcinoma 12% Breast cancer 9%	NH lymphoma 10% Multiple myeloma 5%	47 (22%) were aCL positive, compared with 3 in the control group	NA	Thromboembolic events in 13 patients
Miesbach et al. (2006) [22]	58	59	55	67% solid tumors	NH lymphoma 15% Myeloproliferative diseases 8% Acute leukaemia 3%	LA 48% IgG aCL 40% IgM aCL 62%	3 patients APS associated to SLE *	In patients with solid tumours 46% and 32% in haematological
Font et al. (2011) [23]	258	58	43	Colorectal 24% Lung carcinoma 14% Breast cancer 14% Urinary tract tumors 14%	--	aPL positive 8%	4 patients met APS criteria **	Four patients met classification criteria for APS
Yooon et al. (2003) [24]	33	58	57	Non-Small cell lung cancer 27% Colorectal 15% Ovarian 12%	NH lymphoma 6%	aPL 60% IgA B2GP1 46% aCL IgG 6.7% aCL IgM 16.7%	NA	Venous thrombosis 87% Arterial thrombosis 24%
de Meis et al. (2009) [25]	105	NR	NR	Lung carcinoma 100%	--	In thrombosis group LA 36%, B2GP1 9%	NA	Presence of β2GP1 IgM was negatively correlated with thrombosis
Bazzan et al. (2009) [26]	137	61	73	Breast cancer 56% Colonrectal 16% Head-neck 12%	Haematological disease 11%	Overall aPL 24%, LA 5.8%, aCL IgG 8.8%, aCL IgM 3.6% B2GP1 IgG 3.6%, B2GP1 IgM 2.2%	5 patients met APS criteria **	Nine (6.5%) patients with VTE.
Vassalo et al. (2014) [27]	95	63	44	Solid tumors 79% Gastrointestinal 23% Head and neck 12% Brain 11%	Hematological 21% NH lymphoma 10% Hodgkin’s lymphoma 4%, Acute leukaemia 4%	LA 61% IgA B2GP1 31% aCL 1%	NA	Venous thrombosis in 4% All patients admitted at ICU

* Sapporo APS Criteria; ** Sydney APS Criteria.

## Abbreviations

The following abbreviations are used in this manuscript:

aCL: Anticardiolipin
Anti-β2GP1: Anti-beta 2 glycoprotein 1
aPL: Anti-phospholipid antibodies
APS: Antiphospholipid syndrome
CAPS: Catastrophic Antiphospholipid Syndrome
DVT: Deep vein thrombosis
LA: Lupus anticoagulant
MI: Myocardial infarction
PS: phosphatidylserine
PT: Prothrombin
SLE: Systemic lupus erythematosus
